# Beyond All-Sky: Assessing Ecological Light Pollution Using Multi-Spectral Full-Sphere Fisheye Lens Imaging

**DOI:** 10.3390/jimaging5040046

**Published:** 2019-04-09

**Authors:** Andreas Jechow, Christopher C.M. Kyba, Franz Hölker

**Affiliations:** 1 Ecohydrology, Leibniz-Institute of Freshwater Ecology and Inland Fisheries, 12587 Berlin, Germany; 2 Remote Sensing, GFZ German Research Centre for Geosciences, 14473 Potsdam, Germany; 3 Institute of Biology, Freie Universität Berlin, 14195 Berlin, Germany

**Keywords:** light pollution, artificial light at night, night-time, imaging, radiance measurements, luminance measurements, ecological light pollution

## Abstract

Artificial light at night is a novel anthropogenic stressor. The resulting ecological light pollution affects a wide breadth of biological systems on many spatio-temporal scales, from individual organisms to communities and ecosystems. However, a widely-applicable measurement method for nocturnal light providing spatially resolved full-spectrum radiance over the full solid angle is still missing. Here, we explain the first step to fill this gap, by using a commercial digital camera with a fisheye lens to acquire vertical plane multi-spectral (RGB) images covering the full solid angle. We explain the technical and practical procedure and software to process luminance and correlated color temperature maps and derive illuminance. We discuss advantages and limitations and present data from different night-time lighting situations. The method provides a comprehensive way to characterize nocturnal light in the context of ecological light pollution. It is affordable, fast, mobile, robust, and widely-applicable by non-experts for field work.

## 1. Introduction

The increasing use of artificial light at night (ALAN) [1,2] has resulted in a novel environmental stressor, light pollution, that can appear, for example, as astronomical [3] or as ecological light pollution (ELP) [4,5,6]. Many animals are nocturnal and live in dim light habitats, with some even using visual cues from stars for orientation [7]. ALAN and the subsequent ELP have shown diverse impacts on species level, for example on micro-organisms [8], corals [9], plants [10], insects [11], fish [12,13], birds [14,15], or mammals [16,17]. ELP can also disrupt important ecosystem services such as pollination [18] and can affect whole ecological communities [19] and alter daytime behavior of species [13]. It could furthermore be a driver of evolution [20,21] and affect biodiversity [22], the latter also due to large-range effects of ALAN scattered in the atmosphere appearing as skyglow [1].

However, despite the amount of experimental work on ALAN and ELP, the understanding of the nocturnal light field (natural and artificial) at a specific site under specific environmental conditions (e.g., clouds, snow) is still limited. The natural nocturnal light field changes due to the movement of celestial objects, including daily variation in daylength and moon illumination as well as a seasonal variation in the position of stars [23]. Weather conditions further impose changes on shorter timescales to the natural light field, because atmospheric conditions significantly alter the amount of light incident at the Earth’s surface, and the surface reflectance can change due to changes in vegetation, soil moisture, or snow cover. ALAN and therefore ELP undergo similar dynamics depending on both the usage habit of humans switching lights on and off [24,25] and weather conditions [26,27]. ALAN thus alters intensity, spectrum, and directional patterns of the nocturnal light field. To evaluate ELP in the context of ecologically important visual cues and signals, all these parameters are important to know for in depth ALAN studies. Unfortunately, at the moment no measurement device or method that can provide all these parameters simultaneously at high resolution is available for dim light conditions. However, in the context of astronomical light pollution, all-sky cameras have emerged recently as a promising tool for night sky brightness measurements [23,24,25,26,27,28] (see discussion for more details).

**Figure 1:**
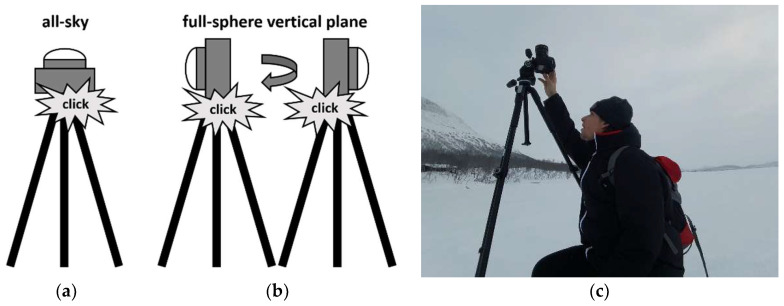
Operating the camera system (**a**) in all-sky mode, (**b**) in vertical 4pi mode and (**c**) during field work under harsh conditions (on Lake Kilpisjärvi, Finland, image by F. Hölker).

Here we explain how to apply fisheye lens photometry with commercial digital cameras to assess ELP more comprehensively. Unlike in all-sky photometry (Figure 1a), where the primary imaging plane is horizontal, we use two vertical plane images in the opposite direction to acquire full sphere 4pi light field information (Figure 1b). This is important as most sources of light pollution are typically located near the horizon where the lens aberrations are highest in all-sky imaging. Furthermore, the information of light emitted from below the horizon including the ground is missing in all-sky imaging. Many animals (and humans) have their viewing direction towards the horizon or the ground rather than up into the sky [29], while few rely on celestial cues [7] or both terrestrial and celestial visual cues in concert [30].

We describe the technical and practical procedure of the method step by step in detail and give examples of two software solutions to analyze the results, to make the method accessible to a broad range of users. Images can be converted to luminance maps and parameters such as scalar illuminance can be extracted from the data. Furthermore, the multi-spectral information can be used to calculate correlated color temperature (CCT) maps. We discuss advantages and current limitations of the method in the context of data from different night-time lighting conditions as well as seasonal variations (snow, clouds). With our protocol, the method can be widely applied by non-experts in photometry or astronomy as it relies on commercial and easy-to-handle equipment and easy-to-use commercial software, or freely available software requiring only minor programming skills.

## 2. Materials and Methods

A calibrated digital camera with a 180° fisheye lens is used to obtain two images back to back in the vertical plane, and an optional all-sky image in the horizontal plane (Figure 1). The raw images of the camera are then translated into luminance maps or optional to CCT maps that can then be used to assess ELP. Here we describe the necessary hardware, software, and give details of the alignment and measurement procedure step by step.

### 2.1. Basic Hardware

The basic setup consists of a digital camera, a circular fisheye lens with 180° field of view, and a tripod. Auxiliary equipment included a remote control, heating pads to avoid dew or ice formation on the lens during cold or humid weather (see e.g. Figure 1c), and a tool to align the camera with respect to the horizon, such as a bull’s eye spirit level. The digital camera must be capable of saving files in an unaltered raw file format, support high ISO format (>3200) and allow long exposure times (e.g., in “bulb mode”). Camera and lens distortions such as vignetting should be known, either by calibrating the system individually (in case of Sky Quality Camera software, see below), or by selecting a camera from a list of supported cameras by the software (in case of DiCaLum software, see below). The most commonly supported cameras are full frame digital single lens reflex (DSLR) cameras, but some mirrorless cameras and DSLRs with smaller frame are supported, too. In principle, it is possible to calibrate almost any camera and lens combination (also homebuilt ones) but there are constraints regarding signal to noise, and we advise the use of commercial consumer electronics for simplicity and inter-comparison of results.

Our camera of choice is a Canon EOS 6D DSLR (Canon Inc., Tokyo, Japan) with a full-frame CMOS sensor with 20.2 Megapixels. Our choice of lens is a circular fisheye lens (Sigma EX DG, Sigma Corporation, Kawasaki, Japan), with an 8 mm focal length and an aperture of 3.5.

### 2.2. Measurement Protocol

#### 2.2.1. Focusing

In the first step, the camera must be focused on a distant object. On clear nights, a bright celestial object such as a planet or a star can be tracked in the live view of the camera. The best procedure is to adjust the lens to infinity first and then carefully focus to shorter distances (normally this is indicated at the lens). It takes some trial and error to find the small point source in the live view but focusing to a small point source is then rather trivial, as “smearing-out” and dimming is obvious. If stars are not visible, for example in cloudy or overcast conditions, focusing can be done using a distant artificial light source (e.g., on the horizon). Once the focus is found, it is possible to fix it with adhesive tape, especially if the lens is only used for such measurements. 

#### 2.2.2. Camera Alignment

##### (a) All-sky Alignment (Optional)

The standard procedure to measure astronomical light pollution is to obtain all-sky images as shown in Figure 1a). To do so, the camera should first be pointed with the lens facing the horizon towards the southern horizon. The camera is then tipped back by 90° to point the lens towards the zenith. This pre-alignment is in principle not necessary but guarantees that south is in the lower part of the image. This makes it much easier to identify distant sources of light pollution, particularly for overcast conditions. A flat lens cap and a bull’s eye spirit level can be used for fine alignment to the horizontal plane.

##### (b) Vertical Alignment

The preferred method to assess ELP is to obtain images in the vertical plane as shown in Figure 1b instead of all-sky images (see Discussion Section). Here, the camera should be aligned with the lens pointing towards the horizon, which can be accomplished with spirit levels or the cameras built in level feature if available. A minimum set of data would consist of two images obtained in opposite directions. In this case, we advise to bring the most interesting or brightest feature in the center of the first image (i.e. the skyglow from a large city) and then simply rotate the camera 180° to obtain the other image. This would be the preferred option when many sites have to be characterized within a short time frame. For time series [24,25], two cameras could be operated back to back. In order to even more carefully characterize a site, it would be possible to obtain a set of four images in the four cardinal directions and a fifth in the all-sky direction.

#### 2.2.3. Aperture Settings

The aperture must be set to the maximum value (3.5 for our fisheye lens) to collect as much light as possible, and because typically the radiometric calibration for the described software is also only available for maximum aperture. However, additional calibration for other aperture settings is in principle possible.

#### 2.2.4. Exposure Times and ISO Settings

Finding the right exposure times and ISO speed setting is important to minimize noise and avoid saturation. Generally, a low ISO is less noisy, but requires very long exposure times. Long exposure times are unpractical for time series, and moving objects such as clouds, and lead to smearing of celestial objects due to the Earth’s rotation. After an initial first shot at a new site, the histogram of the acquired image can be consulted on the camera’s display to avoid saturation or underexposure. The peak of the histogram should be towards the center, reasonably far away from zero, and there should not be an additional peak at the highest values. For a near-natural sky we operated the EOS 6D with ISO 3200 or 6400, and 30 s exposure without using the remote control, or up to 120 s with remote. In urban settings, we used ISO 1600 and down to as short as a 0.5 s exposure. For inexperienced users, and if measurement time is not a limiting factor, it is advised to take multiple images per measurement direction with varied exposure times at fixed ISO.

### 2.3. Software

#### 2.3.1. Calibration, Illuminance and CCT

There are two software choices readily available for light pollution monitoring with the described camera systems, the commercial "Sky Quality Camera" (SQC) software (latest version 1.8.1, Euromix, Ljubljana, Slovenia) and the free tool “DiCaLum” [31]. Both software tools are primarily designed to assess astronomical light pollution (not strong direct illumination, yet) and have a similar working principle, with luminance calculations using the green channel of the camera (that matches photopic vision relatively closely). Photometric calibration is done based on classical astronomical photometry using star brightness and extinction measurements during a night of photometric quality (i.e. stable atmospheric conditions). Lens vignetting is corrected in the laboratory [31]. For a detailed description of calibration procedures see references [28,31,32,33].

The SQC software processes the luminance *L_v_* for each pixel of the camera (luminance is commonly referred to as “brightness” referenced to human vision). From the spatially resolved luminance maps, the software can derive the (“cosine corrected” or “vector”) illuminance *E_v,cos_* in the imaging plane:

$$E_{v,cos}=\int_{0}^{\frac{\pi }{2}}\int_{0}^{2\pi }L_{v}\left(\theta ,\phi \right)sin\theta cos\theta d\phi d\theta ,$$

and the “scalar” illuminance *E_v,scal,hem_* for the imaging hemisphere (no cosine correction):

$$E_{v,scal,hem}=\int_{0}^{\frac{\pi }{2}}\int_{0}^{2\pi }L_{v}\left(\theta ,\phi \right)sin\theta d\phi d\theta .$$

In the equations, *L_v_* is the luminance, θ is the polar angle angle, and Φ is the azimuth angle. For all-sky images, i.e. when imaging in the horizontal plane, *E_v,hor,cos_* is usually termed horizontal illuminance.

**Figure 2:**
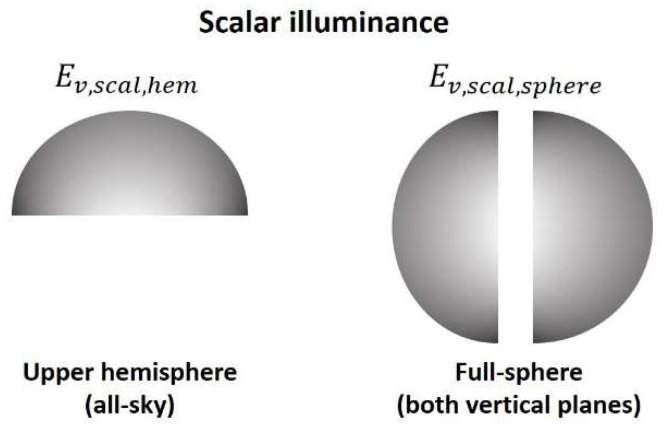
Illustration of hemispherical scalar illuminance obtained from an all-sky image and the total spherical scalar illuminance obtained from two images in the vertical plane.

For ecological light pollution, “scalar” illuminance is a better proxy for the total light incident at a site rather than the commonly measured “vector” or “horizontal” illuminance referenced to the horizontal plane. ”Scalar” illuminance is common in ecology, e.g., aquatic sciences, but please note that from a physical point of view it is equivalent to 2π times the average luminance in the imaging hemisphere. When taking two images in the vertical plane in opposite directions it is possible to acquire the total scalar illuminance *E_v,scal,sphere_* that is the sum of the two individual hemispheric scalar illuminances obtained from each image. This is illustrated in Figure 2.

From the three spectral channels, CCT can be calculated. A transformation from the RGB channels to CIE XYZ color space is done so that it can serve as an indicator for CCT [34]. CCT describes the temperature of a thermal source that most closely matches the color spectrum of the observed light. Candle light has a low CCT (around 2000 K), sunlight has a CCT of about 5800 K, and daylight with a clear sky typically has an even higher CCT as it appears bluish-white.

#### 2.3.2. Sky Quality Camera—SQC

With SQC software, the camera is individually calibrated by the software manufacturer either by purchasing a calibrated camera with the software or by sending a pre-owned camera. SQC features a graphical user interface that is easy to understand and requires no programming skills. SQC calculates luminance maps and CCT maps in circular azimuth projection, cylindrical projection, and in Hammer-Aitoff projection. Furthermore, scalar and in-plane illuminance is automatically extracted. The analysis of a specific direction or a patch of the sky (or the ground) of interest is possible by defining areas [24,27]. When obtaining multiple images, changes in the nocturnal light field can be tracked by subtraction of images [24,25,27] or by batch analysis of changed parameters regarding the entire image or regions of interest. This is particularly useful for temporal analysis with many images [25], but please use care that the camera pointing is identical for multiple images. Two fixed luminance scale bars are available to be compatible with earlier work with CCDs [35]. SQC has additional features mainly tailored for astronomical light pollution studies in all-sky setup (cloud detection, alignment to stars, removal of bright stars, light pollution sources) and to list them all would exceed the scope of this work. SQC allows the user to upload the data to a website portal that has a GIS map interface that can be overlaid with different night-time satellite data [36].

#### 2.3.3. DiCaLum

DiCaLum [37] is a free open source code that relies on a list of cameras and lenses that have been calibrated in the laboratory [31]. The current version uses GNU Octave, a MATLAB compatible computer language and provides luminance maps in natural sky units (NSU) [38], and information about the luminance as a function of the zenith angle. The data acquired with DiCaLum can be further analyzed, and it is possible to obtain information from the other spectral channels [39]. While features like calculating scalar or in-plane illuminance are not built in features, they are easy to retrieve within Octave [26].

A preliminary cross-check between SQC and DiCaLum using several dozens of images ranging from dark sites to urban sites in clear and cloudy conditions showed only a slight deviation between the two software systems, within a few percent.

## 3. Results

We obtained all-sky and vertical plane images at several locations with different weather and seasonal conditions to illustrate several night-time settings with the presence of light pollution: A terrestrial field site near human settlements (Germany, overcast, no snow), an International Dark Sky Park (Hungary, clear sky, no snow), a terrestrial field site in an International Dark Sky Reserve (Germany, cloudy, partial snow cover), a subarctic winter setting (Finland, clear sky, full snow cover), and an aquatic field site in a remote area (Germany, overcast, no snow or ice). The imaging data were processed with the SQC software (see Section 2.3.2).

### 3.1. Lights at and Below the Horizon

#### 3.1.1. Terrestrial Field Site, Marburg, Germany, Cloudy

**Figure 3:**
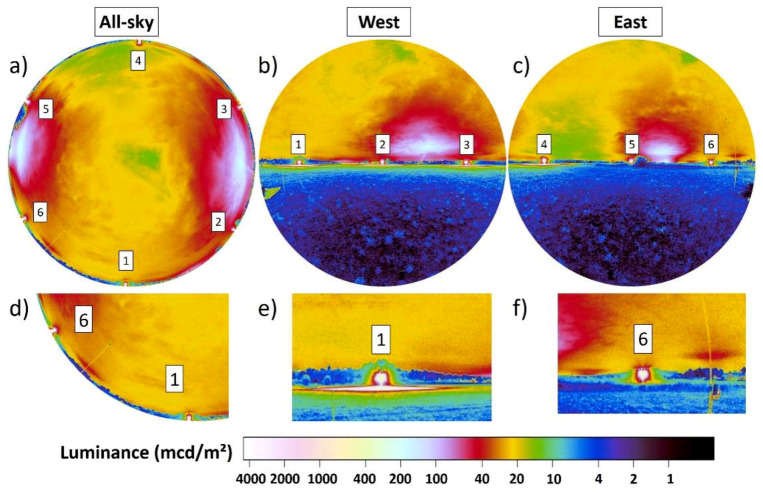
Luminance maps obtained in the center of six sodium vapor street lamps (labelled 1–6) that were arranged in a circle. (**a**) Shows the all-sky data, (**b**) vertical plane data towards the West and (**c**) vertical plane data towards the East. Panels (**d**–**f**) show zoom-in of the luminance maps, to highlight the reflected light just below the horizon. The difference between the two lamps in (**e**) and (**f**) is not perceivable in the all-sky image in (**d**).

**Figure 4:**
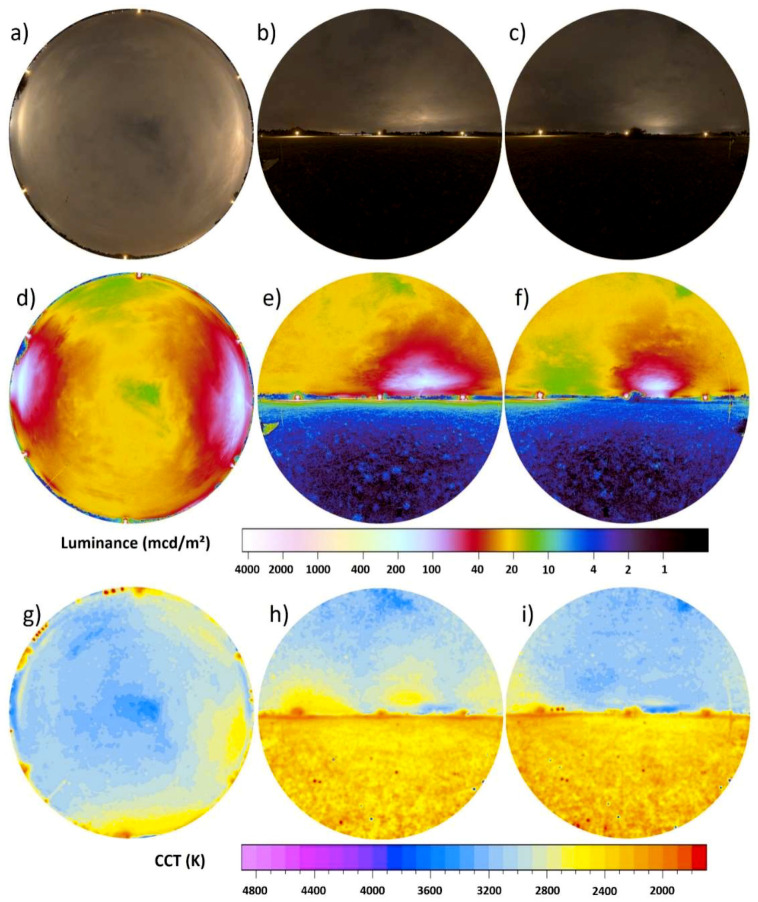
Full data set of Figure 3 with upper row (**a**–**c**) showing RGB images, the center row (**d**–**f**) luminance maps and lower row (**g**–**i**) CCT maps. The left column (**a**,**d**,**g**) shows the all-sky data the middle (**b**,**e**,**h**) and right (**c**,**f**,**i**) columns the vertical plane data.

Figure 3 and Figure 4 show data obtained at the field site near the city of Marburg, Germany. The location of the measurements was at 50°48′48.7″ N 8°52′30.8″ E close to the village Großseelheim. Images were obtained on 12.06.2018 between 00:38 and 00:44 local time (GMT +2) during a night with cloudy to overcast conditions. The camera was positioned in the center of six sodium vapor street lamps arranged in a circle. The purpose of the experiment was to study flight to light behavior of moths [40]. Figure 3 shows the luminance maps for different viewing directions, (a) all-sky, (b) West and (c) East. The six street lamps are numbered, starting at 1 for the southern lamp. The scene is dominated by artificial light, mainly from the nearby six lamps, but also from skyglow amplified by clouds that originated from Marburg to the West (ca. 7 km distance) and the town of Stadtallendorf (ca. 10 km distance). Visual cues from celestial or starlight are not present.

While all six lamps appear almost identical, in the all-sky image (Figure 3a), differences become apparent in the vertical plane images (Figure 3b,c) where information below the horizon is available. Lamps 1, 2, 3, and 4 create a pattern of reflected light from the ground, while lamps 5 and 6 do not (due to bushes and high grass on the ground). This is highlighted in the zoom-ins presented in Figure 3d–f, where details of the two lamps (1 and 6) are depicted. From the camera position, lamp 6 (Figure 3f) does not produce a pattern of reflected light on the ground, whereas lamp 1 (Figure 3e) does. In the all-sky data (Figure 3d) both lamps are indistinguishable.

Figure 4a–i shows the full imaging data with the upper row (a–c) showing RGB images, the middle row (d–f) luminance maps (already presented in Figure 3) and the bottom (g–i) row CCT maps. The left column (a,d,g) shows all-sky, the middle column (b,e,h) the western direction and the right column (c,f,i) the eastern direction. Imaging of the full sphere provides the total scalar illuminance (see Section 2.3.1 and Figure 2). The scalar illuminance from the upper hemisphere was 12.9 mlx derived from the all-sky image (Figure 4c). The vertical data from the West (Figure 4d) gave a hemispheric scalar illuminance of 7.9 mlx and 6.3 mlx from the East (Figure 4e). The sum of the scalar illuminance from the two vertical hemispheric images resulted in a total scalar illuminance of 14.2 mlx, which is about 10% higher than from the all-sky image alone. The ground differs from the sky both in luminance (Figure 4e,f) and CCT (Figure 4h,i). The ground has a lower luminance and is dominated by warm-white colors around 2500 K while the sky has a CCT higher than 3000 K. The horizon is clearly perceptible.

#### 3.1.2. International Dark Sky Park, Zselic, Hungary, Clear Sky

**Figure 5:**
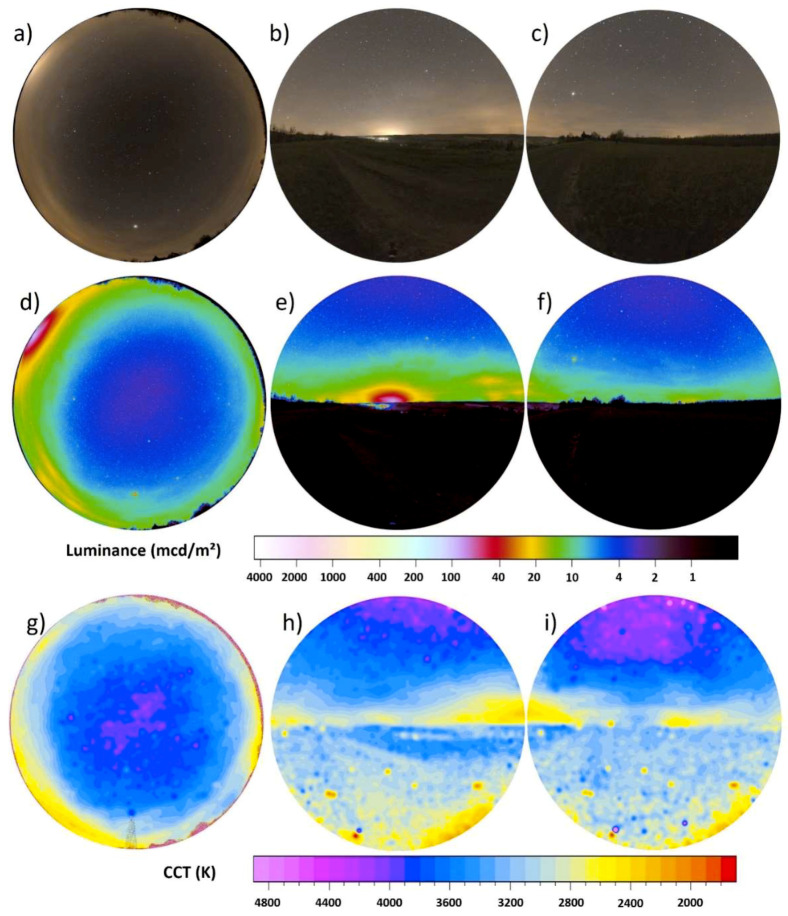
Clear night imaging data Zselic, Hungary. (**a**–**c**) RGB, (**d**–**f**) luminance, (**g**–**i**) correlated color temperature (CCT). Left column (**a**,**d**,**g**) all-sky data, middle (**b**,**e**,**h**) and right (**c**,**f**,**i**) columns vertical plane data.

Figure 5a–i shows the full processed data obtained under clear sky conditions within the International Dark Sky Park Zselic in Hungary (the Zselic National Landscape Protection Area). The measurement position was 46°16′19.1″ N 17°39′49.3″ E and images were obtained on 20.04.2018 between 01:18 and 01:26 local time (GMT +2). The next bigger settlement is the town of Kaposvár to the North at ca. 14 km distance. The upper row (a–c) shows RGB images, the middle row (d–f) the luminance maps and the bottom row (g–i) the CCT maps. Left column shows all-sky, middle column northern direction, and right column southern direction. The skyglow dome from Kaposvár is visible towards the North (middle column), but also a nearby village that lies below the horizon as the data were obtained from a hilltop. The stars as visual cues are visible but still the scenery is dominated by the skyglow (the Milky Way is near the horizon) and an artificial light source below the horizon that is not perceptible in the all-sky image (see Figure 5d,e). During the clear night in Zselic, the ground is much darker than the sky, but the color temperature of skylight and light reflected from the ground is about the same, at least near the horizon. The difference in scalar illuminance is only 6% (3.2 mlx for all-sky and 3.4 mlx for combined vertical).

### 3.2. Snow on the Ground

#### 3.2.1. International Dark Sky Reserve Westhavelland, Partial Snow Cover, Cloudy

**Figure 6:**
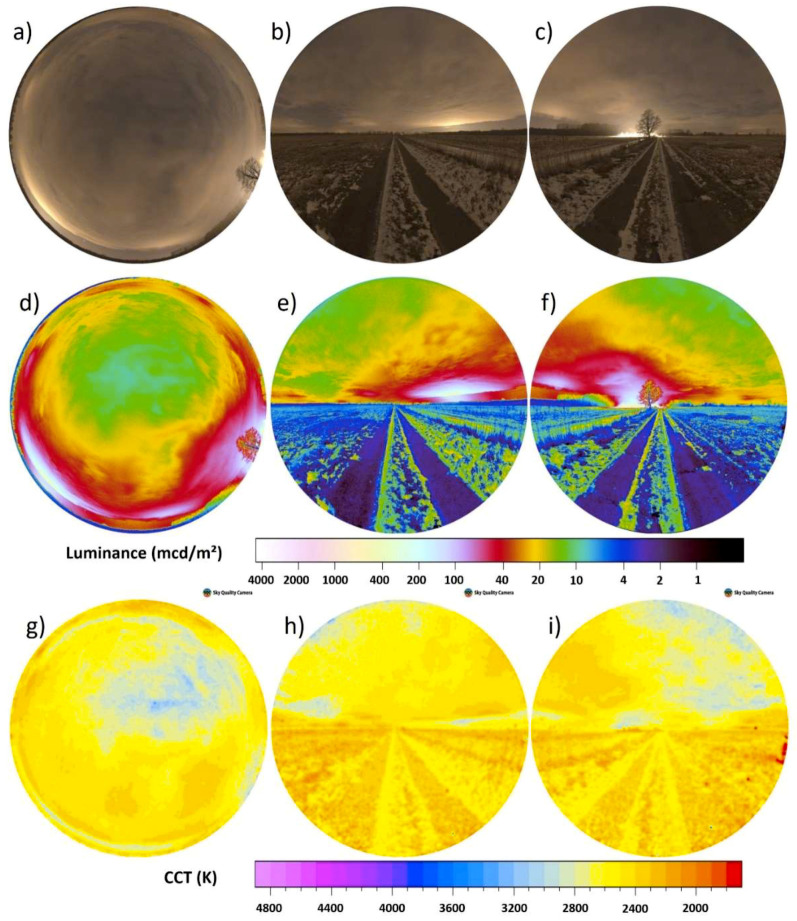
Imaging data taken during an overcast night in winter in the International Dark Sky Park Westhavelland in Germany near an experimental field site. The upper row (**a**–**c**) shows the RGB images, the center row (**d**–**f**) the calculated luminance maps and the lower row (**g**–**i**) the calculated CCT maps. The left column (**a**,**d**,**g**) shows the all-sky data the middle (**b**,**e**,**h**) and right (**c**,**f**,**i**) columns the vertical plane data.

Snow has a very high albedo and, therefore, has a dramatic influence on the amount of light reflected from the ground. Figure 6 shows the data obtained under cloudy conditions and with partial snow cover on the ground within the International Dark Sky Reserve Westhavelland, Germany, near an experimental field site with streetlights [41]. The measurement position was 52°41′29.0″ N 12°27′25.3″ E and images were obtained on 17.01.2017 between 19:38 and 19:46 local time (GMT +1). The next bigger settlements are the town of Rathenow to the Southwest at ca. 15 km distance, the town of Nauen to the Southeast at ca. 30 km distance and the city of Berlin to the Southeast at ca. 70 km distance. The upper row shows RGB images, middle row luminance maps, and the bottom row CCT maps. The left column is all-sky, middle column eastern, and right column western direction.

The dominant visual cues are artificial light sources such as the skyglow from Berlin (Figure 6e) and the lights from the nearby field site (Figure 6f). Both of these are visible in the all-sky image, but the vertical imagery unravels further visual cues such as the road that is not fully covered by snow. The all-sky image gives a scalar illuminance from the upper hemisphere of 11.5 mlx. Towards the East, the hemispheric scalar illuminance was 7.1 mlx and towards the West, the hemispheric scalar illuminance was 7.6 mlx. The sum results in a total scalar illuminance of 14.7 mlx, which is ca. 28% higher than with just all-sky image. Still the horizon can be distinguished in the luminance plots (Figure 6e,f). Both ground and sky are dominated by warm white colors and it is difficult to distinguish sky and ground in the CCT images (Figure 6h,i).

#### 3.2.2. Frozen Lake, Portimo, Finland, Full Snow Cover, Clear

**Figure 7:**
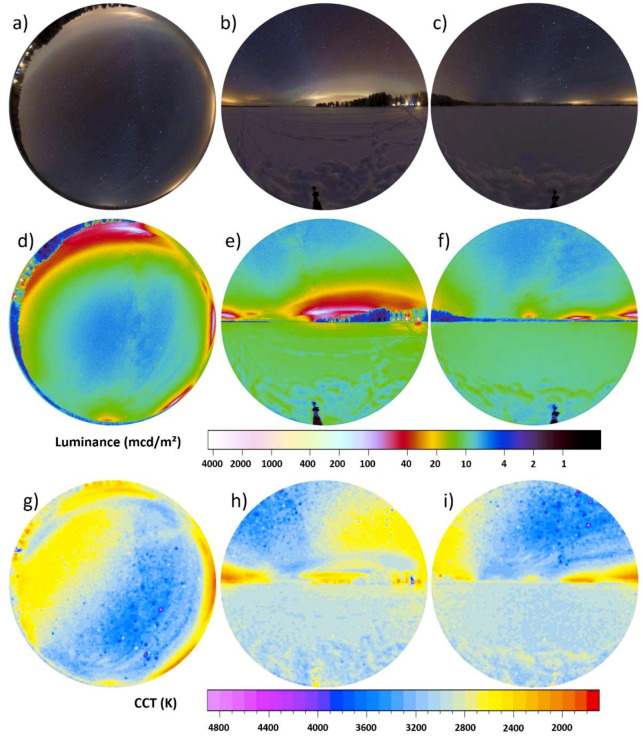
Imaging data taken during a clear night in winter close to the arctic circle in the village of Portimo, Lapland, Finland on a snow-covered frozen lake. (**a**–**c**) RGB, (**d**–**f**) luminance (**g**–**i**) CCT. The left column (**a**,**d**,**g**) is all-sky data, middle (**b**,**e**,**h**), and right (**c**,**f**,**i**) columns vertical plane data.

Figure 7 shows the data obtained under almost clear conditions and with full, freshly fallen, snow cover on the ground on a frozen lake near the arctic circle in Lapland, Finland. The measurement position was 66°05′25.4″ N 26°20′31.5″ E and images were obtained on 02.02.2019 between 21:17 and 21:21 local time (GMT +2). The next bigger settlements are the town of Ranua to the Southeast at ca. 20 km distance and the town of Rovaniemi to the Northwest at ca. 55 km distance. The upper row shows RGB images, the middle row the luminance maps and the bottom row the CCT maps. Left column shows all-sky, middle column eastern direction and right column western direction. An aurora is visible in the northern direction (Figure 7e).

The all-sky data gives a scalar illuminance from the upper hemisphere of 5.3 mlx. Towards the North, the hemispheric scalar illuminance was 5.7 mlx and towards the South, the hemispheric scalar illuminance was 4.0 mlx. The sum from the vertical hemispheric images results in a total scalar illuminance of 9.7 mlx, which is ca. 83% higher than with just the all-sky image, due to the high albedo of the snow. Both ground and sky are dominated by cool white colors with distinct signature of warm white light from artificial light sources near the horizon.

### 3.3. Water Surfaces

**Figure 8:**
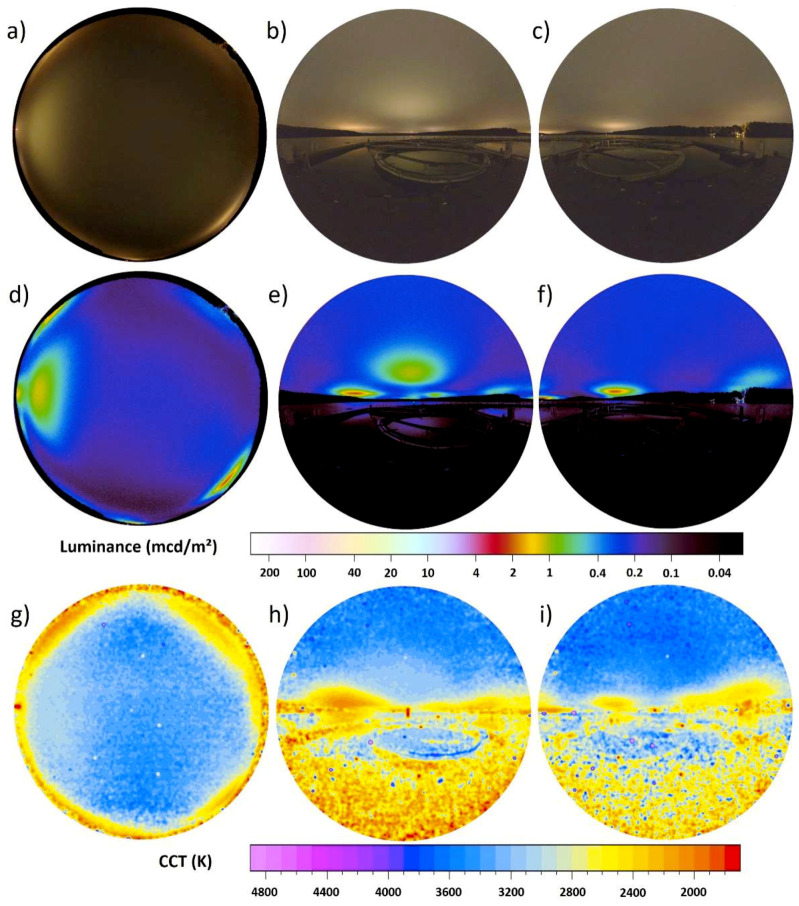
Imaging data taken during an overcast night in summer from a research platform in Lake Stechlin, Germany. The upper row (**a**–**c**) shows the RGB images, the center row (**d**–**f**) the calculated luminance maps and the lower row (**g**–**i**) the calculated CCT maps. The left column (**a**,**d**,**g**) shows the all-sky data the middle (**b**,**e**,**h**) and right (**c**,**f**,**i**) columns the vertical plane data.

The reflectance from still water surfaces depends on the angle of incidence, with shallow angles having a higher reflectivity than steep angles. Figure 8 shows the data obtained under cloudy conditions from the LakeLab, a research platform on Lake Stechlin, Germany. The measurement position was 53°08′34.9″ N 13°01′41.1″ E and images were obtained on 16.08.2016 between 01:22 and 01:26 local time (GMT +2). A more detailed description of the site and surroundings can be found in previous publications [27,39].

The dominant visual cues are artificial light sources, mainly skyglow from nearby villages scattered back from clouds, which is clearly visible in all three images. However, the vertical imagery shows that this skyglow is also reflected at the water surface and by some parts of the floating structure, while other parts of the structure do not reflect much light. This creates further visual cues. Note that the light reflected from the water most likely has a high degree of linear polarization. The all-sky image gives a scalar illuminance from the upper hemisphere of 1.4 mlx. Towards the East, the hemispheric scalar illuminance was 0.7 mlx and towards the West, the hemispheric scalar illuminance was 0.9 mlx. The sum of the scalar illuminance from the vertical hemispheric images results in a total scalar illuminance of 1.6 mlx, which is ca. 14% higher than with just an all-sky image. The sky is dominated by cool white colors with distinct signature of warm white light from artificial light sources near the horizon, while the water surface and ground have more warm-white colors.

## 4. Discussion

Our data shows that the proposed procedure for vertical plane imaging provides valuable additional information compared to all-sky imagery alone. This includes the total amount of light, but also spatial information from the light reflected from the ground, which provides additional visual cues. In this section we briefly discuss other ALAN measurement techniques and describe why the wide angle (fisheye lens) DSLR photometry was chosen in the first place, which alternatives exist, and what a future improved measurement system should look like.

**Satellite remote sensing**: Night-time satellite data are a great tool to assess ALAN at large spatial scales [1,2]. However, satellites measure exclusively the light that is emitted or reflected directly upwards from Earth’s surface. Such data cannot provide information of light propagating upwards at shallow angles, or horizontally or downwards at a specific site. This makes it difficult to derive information about visual cues and signals that are ecologically important for nocturnal species from such data.

**Ground based non-imaging measurements:** Ground based in-situ measurements potentially could provide a higher breadth of information for visual cues compared to remote sensing. However, the parameter that is most commonly assessed by ALAN researchers in ecology is horizontal illuminance [12,42], which provides only limited information on the light field and lacks spatial and spectral information. Furthermore, it either requires expensive devices [42], or suffers from limited sensitivity and precision at low light levels when using standard commercial luxmeters [12]. Spectral irradiance of ALAN can be measured at the Earth’s surface [43,44,45], and with limited resolution even under water [46]. Within astronomy, ground-based observations of the night sky brightness have been established (see review by Hänel et al. [28]) with the most common technique measuring the zenith radiance with a spectrally broadband radiometer like the Sky Quality Meter (SQM, Unihedron, Canada). All these techniques lack directional information, which are important for visual cues.

**Ground based imaging measurements:** The ideal instrument to assess visual cues in the context of ELP would be a hyperspectral imaging system covering the full field of view. Unfortunately, no such system with appropriate sensitivity exists at the moment, and even if it did it would surely be very expensive, preventing its wide application within ELP in the near future. However, a promising pilot study with a hyperspectral camera in a nocturnal urban context was performed recently [47]. CCD cameras with wide angle optics on rotational mounts [37] are the most precise method for astronomical light pollution assessment, but it is time consuming and does not provide color information straightforwardly, limiting its application for ELP. Hänel et al. [28] conclude that the most comprehensive affordable method is to acquire multispectral all-sky images with DSLRs and a fisheye lens. The camera measures the spatially resolved radiance over the whole hemisphere in three spectral bands in a single image. The method is therefore quick, can capture skyglow dynamics [24], and has already been used for field work in freshwater [39], marine [48], and the terrestrial [26] context, as well as under sub-arctic conditions during polar night [49] (see also Figure 1c and Figure 7). All-sky camera systems were first applied in ELP in the context of marine turtles [50] and find more and more recent applications such as celestial cues [7]. Instead of all-sky images, vertical plane images were also obtained but not for the full-sphere [24].

**Advantages of vertical plane images:** There are several reasons to prefer vertical hemispherical images in the context of ELP over all-sky images. First, the fisheye lens system has the highest aberrations (lens distortions) at the edges and although vignetting can be corrected (and is corrected in both software solutions discussed above), the errors at the very edges are higher than in the center of the image [51]. Secondly, many animals (mainly vertebrates and also humans) tend to have their eyes oriented towards the horizon or to the ground to fixate objects [29]. This orientation is used, for example, for foraging, to maneuver around obstacles, to avoid predators, and for social interactions such as communication, reproduction, and group coordination [52]. Third, the information from the ground is missing in all-sky images. One example of a species relying on visual cues at or below the horizon are sea turtle hatchlings finding their way to the ocean [53]. These hatchlings are often disturbed by ALAN [54].

**Figure 9:**
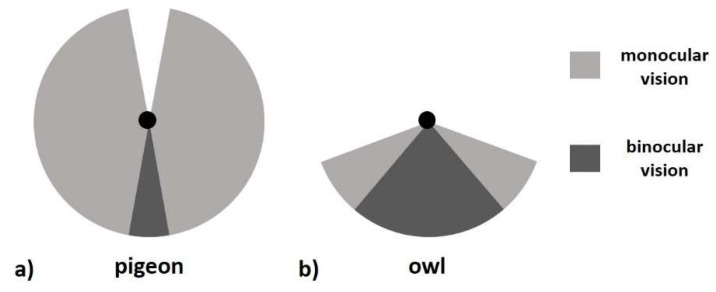
Field of view (in the horizontal plane) of (**a**) a pigeon (*Columba livia*) that scans for predators and (**b**) a tawny owl (*Strix aluco*) looking for prey. Note the differences in total field of view and binocular vision (after [56]).

The difference in field of view can be very large between predators or prey [55], due to the differences in how the eyes are positioned and their different pupil shapes [29]. This is shown in Figure 9 for the example of (a) a pigeon (*Columba livia)* and (b) a tawny owl (*Strix aluco*). To assess what an animal sees in a specific situation, it is best to have the light information from the full-sphere, which can later be reduced to the specific field of view and angular resolution of a specific species [7]. The information from a single point measurement without spatial resolution (like from a luxmeter) is not very useful to predict a specific animal behavior. The ability of an animal to use terrestrial cues for orientation at night is much more dependent on whether the cues have a sufficient contrast against the background than on their own particular luminance [56]. 

When inspecting our data set, it is obvious that some important information about ALAN can come from below the horizon. This includes additional visual cues such as the ground reflection pattern from the lamps in the first dataset (Figure 3 and Figure 4), from structures in the snow (Figure 5), or from water bodies (Figure 8). Each of these details was only visible in the vertical images. Such additional visual cues could result in different behavior of animals. Furthermore, the missing total light when only obtaining all-sky images can vary from ca. 6% (Figure 8) to more than 80% in scalar illuminance for snow on the ground (Figure 7). The additional portion of upwelling light can be in principle be estimated for natural light sources that are incident from large distances, like the moon, when the surface albedo is known. However, as ALAN normally has a very complex spatial distribution, this is almost impossible to infer for light pollution scenarios.

## 5. Conclusions

We have presented a new protocol for night-time fisheye lens photometry for the assessment of ELP, and visual cues in particular. Fisheye lens photometry with commercial digital cameras is the most comprehensive, versatile, and at the same time simple to operate ALAN measurement techniques for site characterizations available at the moment. Unfortunately, the knowledge to apply fisheye lens photometry, which was originally developed in astronomy for horizontal plane imaging to ELP, is fragmented across disciplines and over several individual papers. We channelize this fragmented knowledge and present for the first time a step by step explanation (i) to use fisheye lens photometry for acquiring full solid angle 4pi light field information and (ii) to make this method more accessible to researchers from the life science disciplines that study the impacts of ALAN. Our data shows that the proposed procedure for vertical plane imaging provides valuable additional information compared to all-sky imagery alone. This includes the total amount of light but also spatial information from the light reflected from the ground, which provides additional visual cues that could be linked to different visual systems. A drawback is the currently limited dynamic range, but this method could be extended to high dynamic range (HDR) photography to assess light fields with very high intensity contrast, as recently demonstrated for light assessment regarding humans [57]. Furthermore, the RGB color information tailored to human visual systems of stock digital cameras is limited and many animals perceive nocturnal habitats very differently from humans. In principle, the wavelength range of the method and the number of color channels can be extended, and the color channels tailored to specific needs, as recently demonstrated with seven spectral channels for research in paintings [58]. We hope that this practical tool paper will help to acquire more full sphere night-time imaging data, which will be useful for nocturnal light and ELP studies in the future.

## References

[1] Falchi F., Cinzano P., Duriscoe D., Kyba C.C.M., Elvidge C.D., Baugh K., Portnov B.A., Rybnikova N.A., Furgoni R. (2016). The new world atlas of artificial night sky brightness. Sci. Adv..

[2] Kyba C.C.M., Kuester T., Sanchez de Miguel A., Baugh K., Jechow A., Hölker F., Bennie J., Elvidge C.D., Gaston K.J., Guanter L. (2017). Artificially lit surface of Earth at night increasing in radiance and extent. Sci. Adv..

[3] Riegel K.W. (1973). Light pollution. Science.

[4] Longcore T., Rich C. (2004). Ecological light pollution. Front. Ecol. Environ..

[5] Rich C., Longcore T. (2006). Ecological Consequences of Artificial Night Lighting.

[6] Gaston K.J., Visser M.E., Hölker F. (2015). The biological impacts of artificial light at night: The research challenge. Philos. Trans. R. Soc. B.

[7] Foster J.J., Smolka J., Nilsson D.E., Dacke M. (2018). How animals follow the stars. Proc. R. Soc. Lond. B. Biol. Sci..

[8] Hölker F., Wurzbacher C., Weißenborn C., Monaghan M.T., Holzhauer S.I., Premke K. (2015). Microbial diversity and community respiration in freshwater sediments influenced by artificial light at night. Philos. Trans. Royal Soc. B.

[9] Bhattacharya D., Agrawal S., Aranda M., Baumgarten S., Belcaid M., Drake J.L., Erwin D., Foret S., Gates R.D., Gruber D.F. (2016). Comparative genomics explains the evolutionary success of reef-forming corals. eLife.

[10] Matzke E.B. (1936). The effect of street lights in delaying leaf-fall in certain trees. Am. J. Bot..

[11] Degen T., Mitesser O., Perkin E.K., Weiß N.-S., Oehlert M., Mattig E., Hölker F. (2016). Street lighting: Sex-independent impacts on moth movement. J. Anim. Ecol..

[12] Riley W.D., Bendall B., Ives M.J., Edmonds N.J., Maxwell D.L. (2012). Street lighting disrupts the diel migratory pattern of wild Atlantic salmon, *Salmo salar* L., smolts leaving their natal stream. Aquaculture.

[13] Kurvers R.H.J.M., Drägestein J., Hölker F., Jechow A., Krause J., Bierbach D. (2018). Artificial Light at Night Affects Emergence from a Refuge and Space Use in Guppies. Sci. Rep..

[14] Raap T., Pinxten R., Eens M. (2016). Artificial light at night disrupts sleep in female great tits (*Parus major*) during the nestling period, and is followed by a sleep rebound. Environ. Pollut..

[15] Cabrera-Cruz S.A., Smolinsky J.A., Buler J.J. (2018). Light pollution is greatest within migration passage areas for nocturnally-migrating birds around the world. Sci. Rep..

[16] Kuijper D.P., Schut J., van Dullemen D., Toorman H., Goossens N., Ouwehand J., Limpens H.J.G.A. (2008). Experimental evidence of light disturbance along the commuting routes of pond bats (*Myotis dasycneme*). Lutra.

[17] Robert K.A., Lesku J.A., Partecke J., Chambers B. (2015). Artificial light at night desynchronizes strictly seasonal reproduction in a wild mammal. Proc. R. Soc. Lond. B. Biol. Sci..

[18] Knop E., Zoller L., Ryser R., Gerpe C., Hörler M., Fontaine C. (2017). Artificial light at night as a new threat to pollination. Nature.

[19] Sanders D., Gaston K.J. (2018). How ecological communities respond to artificial light at night. J. Exp. Zool. A.

[20] Swaddle J.P., Francis C.D., Barber J.R., Cooper C.B., Kyba C.C.M., Dominoni D.M., Shannon G., Aschehoug E., Goodwin S.E., Kawahara A.Y. (2015). A framework to assess evolutionary responses to anthropogenic light and sound. Trends Ecol. Evol..

[21] Hopkins G.R., Gaston K.J., Visser M.E., Elgar M.A., Jones T.M. (2018). Artificial light at night as a driver of evolution across urban–rural landscapes. Front. Ecol. Environ..

[22] Hölker F., Wolter C., Perkin E.K., Tockner K. (2010). Light pollution as a biodiversity threat. Trends. Ecol. Evol..

[23] Duriscoe D.M. (2016). Photometric indicators of visual night sky quality derived from all-sky brightness maps. J. Quant. Spectrosc. Radiat. Transf..

[24] Jechow A., Ribas S.J., Domingo R.C., Hölker F., Kolláth Z., Kyba C.C.M. (2018). Tracking the dynamics of skyglow with differential photometry using a digital camera with fisheye lens. J. Quant. Spectrosc. Radiat. Transf..

[25] Jechow A. (2019). Observing the Impact of WWF Earth Hour on Urban Light Pollution: A Case Study in Berlin 2018 Using Differential Photometry. Sustainability.

[26] Jechow A., Kolláth Z., Ribas S.J., Spoelstra H., Hölker F., Kyba C.C.M. (2017). Imaging and mapping the impact of clouds on skyglow with all-sky photometry. Sci. Rep..

[27] Jechow A., Hölker F., Kyba C. (2019). Using all-sky differential photometry to investigate how nocturnal clouds darken the night sky in rural areas. Sci. Rep..

[28] Hänel A., Posch T., Ribas S.J., Aubé M., Duriscoe D., Jechow A., Kolláth Z., Lolkema D.E., Moore C., Schmidt N. (2018). Measuring night sky brightness: Methods and challenges. J. Quant. Spectrosc. Radiat. Transf..

[29] Banks M.S., Sprague W.W., Schmoll J., Parnell J.A., Love G.D. (2015). Why do animal eyes have pupils of different shapes?. Sci. Adv..

[30] Warrant E., Dacke M. (2011). Vision and visual navigation in nocturnal insects. Annu. Rev. Entomol..

[31] Kolláth Z., Dömény A. (2017). Night sky quality monitoring in existing and planned dark sky parks by digital cameras. Int. J. Sustain. Light..

[32] Kocifaj M., Solano Lamphar H.A., Kundracik F. (2015). Retrieval of Garstang’s emission function from all-sky camera images. Mon. Not. R. Astron. Soc..

[33] Falchi F. (2011). Campaign of sky brightness and extinction measurements using a portable CCD camera. Mon. Not. R. Astron. Soc..

[34] Valencia J.S.B., Giraldo F.E.L., Bonilla J.F.V. Calibration method for Correlated Color Temperature (CCT) measurement using RGB color sensors. Proceedings of the Symposium of Signals, Images and Artificial Vision-2013: STSIVA-2013.

[35] Duriscoe D.M., Luginbuhl C.B., Moore C.A. (2007). Measuring Night-Sky Brightness with a Wide-Field CCD Camera. Publ. Astron. Soc. Pac..

[36] Light Pollution Map. https://www.lightpollutionmap.info.

[37] DiCaLum Website. http://dicalum.eu.

[38] Jechow A., Hölker F., Kolláth Z., Gessner M.O., Kyba C.C.M. (2016). Evaluating the summer night sky brightness at a research field site on Lake Stechlin in northeastern Germany. J. Quant. Spectrosc. Radiat. Transf..

[39] Kolláth Z., Dömény A., Kolláth K., Nagy B. (2016). Qualifying lighting remodelling in a Hungarian city based on light pollution effects. J. Quant. Spectrosc. Radiat. Transf..

[40] Degen J., Jechow A., Storms M., Hölker F. Combining radar technology and all-sky imagery to study flight to light behavior of moths. Proceedings of the ALAN Conference.

[41] Holzhauer S.I., Franke S., Kyba C.C.M., Manfrin A., Klenke R., Voigt C.C., Lewanzik D., Oehlert M., Monoghan M.T., Schneider S. (2015). Out of the dark: Establishing a large-scale field experiment to assess the effects of artificial light at night on species and food webs. Sustainability.

[42] Perkin E.K., Hölker F., Heller S., Berghahn R. (2014). Artificial light and nocturnal activity in gammarids. PeerJ.

[43] Spitschan M., Aguirre G.K., Brainard D.H., Sweeney A.M. (2016). Variation of outdoor illumination as a function of solar elevation and light pollution. Sci. Rep..

[44] Secondi J., Dupont V., Davranche A., Mondy N., Lengagne T., Théry M. (2017). Variability of surface and underwater nocturnal spectral irradiance with the presence of clouds in urban and peri-urban wetlands. PLoS ONE.

[45] Ludvigsen M., Berge J., Geoffroy M., Cohen J.H., Pedro R., Nornes S.M., Johnsen G. (2018). Use of an Autonomous Surface Vehicle reveals small-scale diel vertical migrations of zooplankton and susceptibility to light pollution under low solar irradiance. Sci. Adv..

[46] Tamir R., Lerner A., Haspel C., Dubinsky Z., Iluz D. (2017). The spectral and spatial distribution of light pollution in the waters of the northern Gulf of Aqaba (Eilat). Sci. Rep..

[47] Alamús R., Bará S., Corbera J., Escofet J., Palà V., Pipia L., Tardà A. (2017). Ground-based hyperspectral analysis of the urban nightscape. ISPRS J. Photogramm. Remote Sens..

[48] Jechow A., Kolláth Z., Lerner A., Hänel A., Shashar N., Hölker F., Kyba C.C.M. (2017). Measuring light pollution with fisheye lens imagery from a moving boat, a proof of concept. Int. J. Sustain. Light..

[49] Jechow A., Hölker F. Winter (and arctic) light pollution: A new frontier? In Proceedings of the ALAN Conference, Snowbird, UT, USA, 12–14 November 2018.

[50] Thums M., Whiting S.D., Reisser J., Pendoley K.L., Pattiaratchi C.B., Proietti M., Meekan M.G. (2016). Artificial light on water attracts turtle hatchlings during their near shore transit. R. Soc. Open Sci..

[51] Cauwerts C., Bodart M., Deneyer A. (2012). Comparison of the vignetting effects of two identical fisheye lenses. Leukos.

[52] Kurvers R.H., Hölker F. (2014). Bright nights and social interactions: A neglected issue. Behav. Ecol..

[53] Oliver L.J., Salmon M., Wyneken J., Hueter R., Cronin T.W. (2000). Retinal anatomy of hatchling sea turtles: Anatomical specializations and behavioral correlates. Mar. Freshw. Behav. Physiol..

[54] Bourgeois S., Gilot-Fromont E., Viallefont A., Boussamba F., Deem S.L. (2009). Influence of artificial lights, logs and erosion on leatherback sea turtle hatchling orientation at Pongara National Park, Gabon. Biol. Conserv..

[55] Martin G.R. (1984). The visual fields of the tawny owl, *Strix aluco* L.. Vis. Res..

[56] Kaul R.M., Kopteva G.A. (1982). Night orientation of ants Formica rufa (Hymenoptera: Formicidae) upon movement on routes. Zool. Zhurnal.

[57] Jung B., Inanici M. (2018). Measuring circadian lighting through high dynamic range photography. Light. Res. Technol..

[58] Colantonio C., Pelosi C., D’Alessandro L., Sottile S., Calabrò G., Melis M. (2018). Hypercolorimetric multispectral imaging system for cultural heritage diagnostics: An innovative study for copper painting examination. Eur. Phys. J. Plus.

